# Impact of shear stress and atherosclerosis on entrance-tear formation in patients with acute aortic syndromes

**DOI:** 10.1007/s00380-013-0328-z

**Published:** 2013-03-10

**Authors:** Eiji Taguchi, Kazuhiro Nishigami, Shinzo Miyamoto, Tomohiro Sakamoto, Koichi Nakao

**Affiliations:** Division of Cardiology, Saiseikai Kumamoto Hospital Cardiovascular Center, 5-3-1 Chikami, Minami-ku, Kumamoto, 861-4193 Japan

**Keywords:** Acute aortic syndrome, Shear stress, Etiology, Atherosclerosis

## Abstract

Weak aortic media layers can lead to intimal tear (IT) in patients with overt aortic dissection (AD), and aortic plaque rupture is thought to progress to penetrating atherosclerotic ulcer (PAU) with intramural hematoma (IMH). However, the influences of shear stress and atherosclerosis on IT and PAU have not been fully examined. Ninety-eight patients with overt AD and 30 patients with IMH and PAU admitted to our hospital from 2002 to 2007 were enrolled. The greater curvatures of the aorta, including the anterior and right portions of the ascending aorta and anterior portion of the aortic arch, were defined as sites of high shear stress. The other portions of the aorta were defined as sites of low shear stress based on anatomic and hydrodynamic theories. Aortic calcified points (ACPs) were manually counted on computed tomography slices of the whole aorta every 10 mm from the top of the arch to the abdominal bifurcation point. IT was more often observed at sites of high shear stress in overt AD than in PAU (73.5 vs 20.0 %, *P* < 0.0001). Significantly more ACPs were present in PAU than in overt AD (18.6 ± 8 vs 13.3 ± 10, *P* = 0.007). The present study suggests that high shear stress and less severe atherosclerosis could induce the occurrence of an IT, thereafter progressing to overt AD, and that low shear stress and more severe atherosclerosis could proceed to PAU with IMH. These findings may help to identify the entrance-tear site.

## Introduction

**Fig. 1:**
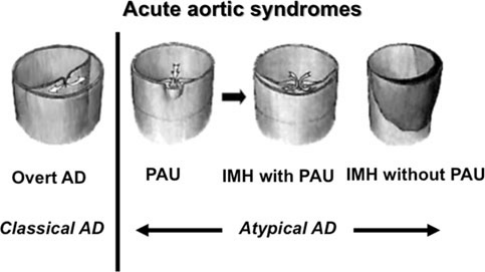
Current concepts of acute aortic syndromes. *AD* aortic dissection, *PAU* penetrating atherosclerotic ulcer, *IMH* intramural hematoma

Acute aortic syndromes include overt aortic dissection (AD), intramural hematoma (IMH), and symptomatic penetrating atherosclerotic ulcer (PAU) [1]. In the classic sense, acute AD requires an aortic intimal tear (IT). Blood passes through the tear, separating the intima from the media or adventitia, resulting in so-called overt AD. PAU is thought to be formed secondary to aortic plaque rupture, which could cause an IT or aortic rupture. IMH has two different etiologies: hemorrhage of the vasa vasorum (IMH without PAU) and thrombosed AD caused by the progression of PAU (IMH with PAU) [2, 3] (Fig. 1). IMH with PAU is significantly associated with a progressive disease course, whereas IMH without PAU typically has a stable course when limited to the descending thoracic aorta [4].

**Fig. 2:**
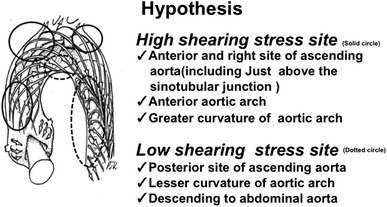
Our hypothesis from the viewpoint of the bloodstream. Schematic drawing illustrates typical development of aortic arch flow [14]

Many factors may be related to entrance-tear formation in acute aortic syndromes, including aortic root motion [5], the geometry of the aorta [6], wall stress [7], intrinsic aortic tissue abnormalities [8], and aortic plaque rupture. For example, entry tears in type A AD are commonly observed just above the sinotubular junction [5] or aortic isthmus portion, near the top of the arch or just before the innominate artery [9]. However, detailed investigation of the entrance-tear formation in acute aortic syndromes has not been performed. We hypothesized that IT tends to occur at sites of high shear stress and that PAU tends to occur at sites of low shear stress. These hypotheses are based on the thought that high shear stress could cause torsion of the IT, leading to IT based on aortic medial degeneration, and that low shear stress could cause aortic plaque formation, leading to plaque rupture from the viewpoint of the bloodstream (Fig. 2). The purpose of the present study was to assess the relationship of shear stress and atherosclerosis with the formation of entrance-tear in patients with acute aortic syndromes.

## Patients and methods

### Study population

A total of 208 consecutive patients admitted to our institution from 2002 to 2007 with diagnoses of acute aortic syndromes were retrospectively analyzed. Of these patients, 98 (50 male and 48 female; mean age, 58 years) with overt AD (double-barrel type AD) and 30 (20 male and 10 female; mean age 68 years) with IMH with PAU were enrolled in this study. Forty-nine patients with IMH without PAU and 31 patients with overt AD in whom the entry site could not be determined were excluded.

Assessment of the entry site in acute aortic syndromes had been assessed by the operative findings, contrast-enhanced computed tomography (CT), and transesophageal echocardiography.

### Shear stress based on blood flow dynamics and anatomy of the aorta

The aorta is thought to possess a curved lumen and flow patterns associated with its structure according to three-directional magnetic resonance velocity mapping [13]. The greater curvatures of the aorta, including the anterior and right portions of the ascending aorta and the anterior portion of the aortic arch, are defined as sites of high shear stress. The other portions of the aorta are defined as sites of low shear stress, based on anatomic and hydrodynamic theories [10, 13]. The blood flow volume can also influence shear stress [10]. The portion from the descending aorta to the abdominal aorta can be defined as sites of low shear stress because the blood flow volume decreases after the aortic arch.

### Atherosclerotic degree of the aorta

**Fig. 3:**
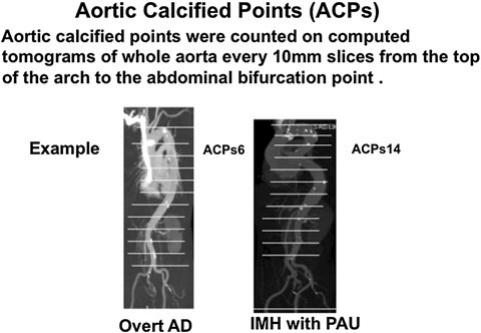
Aortic calcified points, manually counted, constituted the original indicator

To determine the atherosclerotic degree of the aorta, we examined the total number of aortic calcified lesions on CT. Aortic calcified points (ACPs) on calcified lesions were counted on CT slices of the whole aorta every 10 mm from the top of the arch to the abdominal bifurcation point. The ACPs are our original indicator, which were manually counted. If the calcified lesion was identified in one slice, we added 1 point despite the degree or spread of calcification (Fig. 3).

### Statistical analysis

We examined the relationship between shear stress and entrance-tear sites of IT and PAU, and compared the entry site and the atherosclerotic degree between patients with overt AD and patients with IMH and PAU using Student’s *t* test. Values of *P* < 0.05 were considered significant. JMP6.0.3 software (SAS Institute, Cary, NC, USA) was used for statistical analysis.

## Results

The characteristics of the patients are shown in Table 1. We compared the overt AD group and the IMH with the PAU group with respect to age, gender, and atherosclerotic factors including hypertension, diabetes mellitus, smoking, dyslipidemia, and prior myocardial infarction. Age and frequency of prior myocardial infarction were significantly different between the two groups, although the other atherosclerotic factors were not significantly different (*P* < 0.01).

**Table 1:** Baseline characteristics of patients

	Overt AD (*n* = 98)	IMH with PAU (*n* = 30)	*P*
Age (years)	67.8 ± 14.2	73.4 ± 9.7	0.0161
Male sex (*n*, %)	55 (56 %)	12 (40 %)	0.1218
Hypertension (*n*, %)	94 (96 %)	28 (93 %)	0.6244
Diabetes mellitus (*n*, %)	5 (5 %)	4 (13 %)	0.2125
Current smoker (*n*, %)	13 (13 %)	5 (17 %)	0.6391
Ever smoker (*n*, %)	25 (26 %)	11 (37 %)	0.2344
Prior MI (*n*, %)	6 (6 %)	6 (20 %)	0.0225
Total-C (mg/dl)	177.9 ± 38.6	193.2 ± 44	0.1035
HDL-C (mg/dl)	45.0 ± 11.4	50.2 ± 13.0	0.0643
TG (mg/dl)	102.9 ± 56.3	112.1 ± 87.1	0.6039
LDL-C (mg/dl)	112.3 ± 31.5	120.7 ± 31.4	0.2257

*AD* aortic dissection, *PAU* penetrating atherosclerotic ulcer, *IMH* intramural hematoma, *C* cholesterol, *LDL* low-density lipoprotein, *HDL* high-density lipoprotein, *TG* triglycerides

The entrance-tear points in both groups are shown in Table 2. In overt AD, entrance tears were frequently seen in the anterior portion of the ascending aorta and the area just after the left subclavian artery. In IMH, entrance tears were frequently seen in the area just after the left subclavian artery and in the descending aorta.

**Table 2:** Entry portion in both groups

	Overt AD (*n* = 98) (%)		IMH with PAU (*n* = 30) (%)	
High shearing stress				
Anterior site of ascending aorta (*n*, %)	39	40.20	2	6.60
Anterior site of proximal aortic arch (*n*, %)	4	4	1	3.30
Greater curvature of distal aortic arch (*n*, %)	29	29.50	3	10.00
Low shearing stress				
Posterior site of ascending aorta (*n*, %)	6	6.10	3	10.00
Lesser curvature of aortic arch (*n*, %)	6	6.10	1	3.30
Descending aorta (*n*, %)	14	14.20	20	66.66

The relationship between shear stress and the entry point in both groups is shown in Table 3. As mentioned earlier, all points were divided into sites of high shear stress and low shear stress. The analysis showed that the entry point was more frequently demonstrated at sites of high shear stress in overt AD (73.5 %) in contrast to IMH with PAU (20.0 %), with a significant difference (*P* < 0.0001). Table 4 shows that the mean number of ACPs was significantly higher in IMH with PAU than in overt AD, with a *P* value of 0.007 (18.6 ± 8 vs 13.3 ± 10).

**Table 3:** Summary of the site of tear

	Intimal tear portion in overt AD groups	PAU in IMH with PAU groups	*P*
Site of high shearing stress (*n*, %)	72 (73.5 %)	6 (20.0 %)	
Site of low shearing stress (*n*, %)	26 (26.5 %)	24 (80.0 %)	
			<0.0001

**Table 4:** Mean scores of aortic calcified points

	Overt AD (*n* = 98)	IMH with PAU (*n* = 30)	*P*
Aortic calcified points			
Numbers, mean ± SD	13.3 ± 10	18.6 ± 8	0.007

## Discussion

The present study demonstrated that less severe atherosclerosis and higher shear stress may tend to induce the occurrence of an IT that might progress to overt AD, and that more severe atherosclerosis and lower shear stress may tend to induce the development of IMH with PAU. It has been well established that wall shear stress (tangential stress) is an important determinant of endothelial cell function and gene expression as well as of the structure of the wall [10]. Wall stress may be the outcome of several factors, such as the wall components, the geometry of the aorta, the shape of the aneurysm, and the dynamic interaction of the wall with fluid flow [11]. In addition, various measures of aortic stiffness have been proposed as cardiovascular risk markers, but interest has now shifted to more direct and easier evaluation of aortic function [12].

AD has been believed to begin with the formation of a tear in the aortic intima that directly exposes the underlying diseased medial layer to the driving force of intraluminal blood. Shear forces may lead to further tears in the intima. However, few studies have been performed on the relationship between shear stress and IT sites in the clinical setting. The present study disclosed that IT tended to occur at sites of high shear stress in the aorta, including the greater curvatures. These findings may be compatible with the hydrodynamic theory and may help to estimate the entry site.

Ulcer-like projections (ULPs) are easy to confuse with PAU. On aortography, ULPs show a thrombosed false lumen with an intimal defect. They were named on the basis of a characteristic contrast media filling defect that is projected in the shape of an ulcer toward the luminal dissection from the lining membrane. Diagnosis of AD by aortography is more commonly achieved by CT than by aortography. The term ULP is not used in Europe or the United States, but many papers have used the term PAU to describe the flow of contrast media into a pseudolumen. There are currently no criteria to clearly differentiate between ULP and PAU. In Japan, there is a tendency to view ULP as an ulcerative phenomenon accompanying a comparatively wide range of thrombosed-type AD with IT, and to view PAU as more focal thrombosed AD without IT. However, in the present study we did not use the term ULP but the terms IMH with PAU and IMH without PAU, from the perspective of current international standards.

IMH, PAU, and other comorbid diseases associated with atypical AD have increased in the past several years. Atherosclerosis may influence the occurrence of these diseases. ACPs in this study indirectly indicated the severity of atherosclerosis. We were able to demonstrate the relationship between the occurrence of PAU and the degree of ACPs.

Low shear stress has been known to lead to atheroma. PAU has been thought to originate from plaque ruptures on the aortic wall and to cause cholesterol embolism, leading to blue toe syndrome. The present study demonstrated that the occurrence of PAU may be associated with low shear stress in the aorta through severe atherosclerotic risk factors including dyslipidemia, but we failed to demonstrate the relationship between PAU and IT. Further studies are required to elucidate this relationship.

If AD caused by plaque rupture on the aortic wall progresses, IMH with PAU may result. IMH without PAU is thought to occur due to hemorrhage of the vasa vasorum of the aortic wall, and could progress to IT. IT generally causes overt AD with re-entry. If re-entry is absent, as in retrograde dissection, the false lumen is thrombosed, which might look like IMH with PAU.

## Study limitations

There were three limitations in the present study. First, the definition of aortic wall shear stress in vivo has not been established. Although several studies on the influences of wall shear stress have been performed in vitro (Poiseuille’s law) [15], details have not been assessed in vivo. We hypothesized that the greater curvature can be defined as a site of high shear stress and the lesser curvature can be defined as a site of low shear stress, based upon this theory. The blood flow volume can also influence shear stress. The descending abdominal aorta can be defined as a site of low shear stress because the blood flow volume is reduced after the aortic arch. Second, the ACPs counted manually may not have represented the exact degree of the atherosclerosis in the aorta. Atherosclerosis includes the number, depth, length, and quality of plaques. It is difficult to assess these factors in the whole aorta. Third, we were unable to assess the morphology of PAU in this study. It may be crucial to investigate the relationship between the morphology of PAU and the progression of disease prospectively.

## Conclusions

The present study suggests that high shear stress and less severe atherosclerosis could induce the occurrence of IT, which might then progress to overt AD, and that low shear stress and more severe atherosclerosis could induce the progression of PAU with IMH. These findings may help to identify the entrance-tear site in patients with acute aortic syndromes.
